# Unraveling Honey Bee–*Varroa destructor* Interaction: Multiple Factors Involved in Differential Resistance between Two Uruguayan Populations

**DOI:** 10.3390/vetsci7030116

**Published:** 2020-08-20

**Authors:** Yamandú Mendoza, Ivanna H. Tomasco, Karina Antúnez, Loreley Castelli, Belén Branchiccela, Estela Santos, Ciro Invernizzi

**Affiliations:** 1 Sección Etología, Facultad de Ciencias, Universidad de la República, Iguá 4225, Montevideo 11400, Uruguay; ymendozaspina@gmail.com (Y.M.); estelsantos@gmail.com (E.S.); 2 Laboratorio de Evolución, Facultad de Ciencias, Universidad de la República, Iguá 4225, Montevideo 11400, Uruguay; ivanna@fcien.edu.uy; 3 Departamento de Microbiología, Instituto de Investigaciones Biológicas Clemente Estable, Av. Italia 3318, Montevideo 11600, Uruguay; kantunez03@gmail.com (K.A.); castelli.loreley@gmail.com (L.C.); 4 Sección Apicultura, Programa de Producción Familiar, Instituto Nacional de Investigación Agropecuaria La Estanzuela, Ruta 50 km 11, Colonia 70002, Uruguay; bbranchiccela@inia.org.uy

**Keywords:** honey bees, mites, viruses, behavior, social immunity, Africanized bees, microsatellites, Uruguay

## Abstract

The ectoparasite *Varroa destructor* is the greatest biotic threat of honey bees *Apis mellifera* in vast regions of the world. Recently, the study of natural mite-resistant populations has gained much interest to understand the action of natural selection on the mechanisms that limit the mite population. In this study, the components of the *A. mellifera*–*V. destructor* relationship were thoroughly examined and compared in resistant and susceptible honey bee populations from two regions of Uruguay. Mite-resistant honey bees have greater behavioral resistance (hygienic and grooming behaviors) than susceptible honey bees. At the end of the summer, resistant honey bees had fewer mites and a lower deformed wing virus (DWV) viral load than susceptible honey bees. DWV variant A was the only detected variant in honey bees and mites. Molecular analysis by Short Tandem Repeat showed that resistant honey bees were Africanized (*A. m. scutellata* hybrids), whereas susceptible honey bees were closer to European subspecies. Furthermore, significant genetic differentiation was also found between the mite populations. The obtained results show that the natural resistance of honey bees to *V. destructor* in Uruguay depends on several factors and that the genetic variants of both organisms can play a relevant role.

## 1. Introduction

The shift of the ectoparasitic mite *Varroa destructor* from the Asian honey bee *Apis cerana*, its first host, to *Apis mellifera* and its subsequent dispersal throughout the world, has created one of the largest biotic threats to honey bee populations and caused great damages to the beekeeping industry [1,2]. In *A. cerana*, the mite reproduces only in drone cells and it maintains a stable relationship with the host without causing significant damage [3,4,5]). However, in *A. mellifera* it can also reproduce in worker cells and might cause the death of the colonies if acaricides are not regularly applied [1].

In addition to the direct damage caused by *V. destructor* to *A. mellifera*, especially in the brood during the reproductive phase, it also acts as a vector for different RNA viruses and suppresses the immune response of honey bees [6,7]. One of the honey bee viruses that has received more attention due to its close association with *V. destructor* and the colony collapse is the deformed wing virus (DWV) [8,9].

Although *V. destructor* is currently the major threat for honey bees, the damage it causes to populations in different regions around the world varies significantly. For example, the mite has devastating effects in European countries, North America, and temperate regions of South America (Argentina, Chile, and Uruguay), where beekeepers must systematically use synthetic acaricides [2,10,11]. However, in tropical areas of South and Central America and broad regions of Africa, honey bees coexist with the mite without significant problems [11,12]. This difference in the impact of *V. destructor* depends on the time that both species have been interacting (<50 years in most countries), genetic aspects of both honey bees and mites, and the presence of other pathogens, particularly viruses [1,7,13]. Furthermore, the relationship between honey bees and *V. destructor* is strongly influenced by beekeeping practices. Thus, professional beekeepers group colonies into apiaries, which facilitates the horizontal transmission of mites and favors the selection of the most virulent variants [14,15,16]. Other common beekeeping practices, such as honey bee selection, the use of acaricides, movement of colonies, and honey bee trade, can have a strong impact on the interaction between honey bees and mites [15].

The resistance mechanisms of honey bees to *V. destructor* have been widely studied since the 1980s, focusing on hygienic and grooming behaviors [1,17,18,19]. Hygienic behavior (uncapping cells that contain dead, diseased, or parasitized brood and their subsequent removal) is a social behavior that helps control diseases of the offspring such as the American foulbrood (*Paenibacillus larvae*) and Ascosferiosis (*Ascosphaera apis*) and may interrupt the reproduction of *V. destructor* [20]. The selection of honey bees with improved hygienic behavior has had encouraging results in the control of mite populations [18,19]. In the USA, hygienic honey bees have been selected with a particular capacity to detect pupae parasitized by *V. destructor*, a trait known as Varroa Sensitive Hygiene (VSH) [21]. Grooming behavior, by which parasited honey bees can dislodge mites by themselves (autogrooming) or receiving help from other insects (allogrooming) [17], has been reported as an effective resistance mechanism against *V. destructor* [22,23,24,25]. Both hygienic and grooming behaviors are expressed very efficiently in *A. cerana* and would be key to controlling *V. destructor* populations [5,26]. In *A. mellifera*, these behaviors are expressed more highly in Africanized honey bees (hybrids of *A. m. scutellata*) than in European ones, which could partially explain the resistance to *V. destructor* in the former [13].

For several years, researchers from different countries have been working on the breeding and selection of *V. destructor*-resistant honey bees [18,19]. Although significant progress has been made, its impact on the beekeeping industry has been very limited and a situation where beekeepers can maintain their colonies without acaricide treatment is far from being reached. However, the possible successes of these initiatives are still under debate [27,28].

Given the limitations of artificial selection, the study of honey bee populations naturally resistant to *V. destructor* has gained increasing attention in recent years. The most studied populations have been those from Brazil, South Africa, Fernando de Noronha island (Brazil), Gotland island (Sweden), Avignon (France), and Arnost Forest (USA) [13]. It is interesting to observe how natural selection has shaped different responses in different honey bee populations to coexist with *V. destructor*. For example, in Brazil and South Africa, as well as in Primorsky’s honey bees, behavioral resistance is important, whereas in the case of Gotland’s bees, resistance is associated with a reduction in colony size and lower mite reproduction [13].

Unlike honey bees, differences in *V. destructor* that may affect the relationship with its host are less known. Among the several described haplotypes of *V. destructor,* Japanese and Korean are the only ones able to reproduce in *A. mellifera.* It is known that the Korean haplotype display a higher virulence and a wider geographic distribution than the Japanese one [29,30,31]. Strikingly, both haplotypes have almost no polymorphism and can, therefore, be considered as quasi-clonal populations [31]. However, genetic differences between mites at the population and colony levels, even within the colony, have been recently reported [32,33].

Uruguay is a South American country of 176,000 km^2^ without significant geographical barriers, with a temperate climate where the spring–summer period presents marked differences compared to the autumn–winter period. In 1834, *A. m. mellifera* was introduced from France [34], but today most of the honey bee populations are hybrids with *A. m. scutellata* after decades of this subspecies entering from Brazil [35,36]. *Varroa destructor* entered the country in 1978 and until the late 1990s it did not cause significant problems, and colonies were able to survive without acaricide treatments. Afterward, colony losses associated with *V.* destructor increased and, in a few years, beekeepers had to use massively synthetic acaricides in almost all the country in order to ensure colony survival [37]. This change could be due to the introduction of more susceptible European honey bee subspecies, the entry of more virulent variants of *V. destructor*, a greater impact of viruses associated with the mite, among other factors. However, on the eastern side of the country there are still regions where honey bees coexist with *V. destructor* without acaricide treatments and with minimal colony losses [37]. It should be noted that in Uruguay only the K haplotype of *V. destructor* is present [38]. This is a striking situation considering the short distance where susceptible honey bees are found, and the increasing movement of colonies between regions. Thus, this is an interesting scenario to analyze the factors involved in differential resistance to *V. destructor* in Africanized bees.

The aim of this study was to analyze the role of different factors that could affect the *A. mellifera*–*V. destructor* interactions in two populations with marked mite resistance differences.

## 2. Materials and Methods

### 2.1. Overview

During the spring of 2013, one apiary comprising 21 colonies was installed in the experimental station of INIA La Estanzuela (34°20′48.60″ S; 57°41′29.02″ W), Colonia Department (western region of Uruguay), and another apiary with 23 colonies was installed in the experimental station INIA Treinta y Tres (33°15′06.60″ S; 54°25′40.63″ W), in the Treinta y Tres Department (eastern region of Uruguay). In both cases, colonies belonged to local honey bee populations with new queens and no symptoms of disease. Honey bee populations from Colonia received acaricide treatment at least once a year to survive the infestation by *V. destructor* (“mite-susceptible colonies”). On the contrary, colonies located in Treinta y Tres had not received acaricides for six years, showing average infestation levels lower than 5% and low annual colony losses (<15%, “mite-resistant colonies”).

From the middle of the summer of 2014 (January) to the beginning of the autumn of 2014 (April), the evaluations and sampling described below were carried out in both apiaries. Later, in the summer of 2015, the estimation of mites that reproduced in drone and worker cells was evaluated.

### 2.2. Estimation of the Honey Bee Population and Brood Area

In order to estimate the honey bee population at the end of the summer (March), the number of frames covered by honey bees was recorded [39]. In the case of brood, the brood area occupied per frame was estimated [39]. During the winter, the apiaries were regularly visited to determine the survival of colonies.

### 2.3. Evaluation of Hygienic and Grooming Behaviors

The evaluation of hygienic behavior was carried out in 21 mite-susceptible colonies and 21 mite-resistant colonies. At least 100 pupae were killed by pricking them with an entomological pin through the cell cap and 24 h later the number of removed pupae was recorded. The result was expressed as a percentage of cleaned cells [40].

Grooming behavior was evaluated in 17 and 21 mite-susceptible and mite-resistant colonies, respectively. A petroleum jelly-smeared sheet was placed on the floor of the hives for 7 days so that the mites dislodged by the bees would remain attached. Mites were observed at 40× to determine if they had mutilated legs. Grooming behavior was expressed as a percentage of damaged mites [41].

### 2.4. Estimation of Mites in Honey Bees and in Brood Cells

The estimation of the mites’ proportion in honey bees (phoretic mites) and in the brood cells was made in 21 mite-susceptible colonies and 21 mite-resistant colonies.

To estimate the percentage of mite-infected honey bees, a sample of approximately 300 workers collected in three combs was taken from each colony. The varroa mites were removed from the honey bees with ethanol 75%, and the percentage of infected honey bees was determined [42].

The brood infestation was determined by observing 400 capped cells with pupae older than 15 days of age (with purple eyes). When an infested cell was found, the number of adult mites (female founders) and offspring were recorded. Adult mites were differentiated from the immature forms (protonymph and deutonymph) by size, shape, and color. From these records, the fertility of the mites (cells infested with one varroa with offspring), the abundance (the average number of adult female mites per examined cells), the intensity (the average number of female mites per infested cell), and the prevalence (the percentage of infested cells) were estimated. To determine the fertility of *V. destructor*, only colonies with at least 10 infected pupae were considered. Thus, 21 mite-susceptible colonies and 11 mite-resistant colonies were analyzed. The relationship between the infestation level in adult bees and brood (abundance) was determined.

At the end of the summer of 2015, 8 mite-susceptible colonies and 5 mite-resistant colonies, the only ones that had at least 10 drone cells, were selected. None of these colonies had participated in the study carried out the previous year. The presence and quantity of founder females in the cells of drones and workers were recorded to determine the preference of mites to reproduce in the two types of cells.

### 2.5. Detection and Quantification of RNA Viruses in Honey Bees and Mites

Nurse honey bee samples were collected from the brood nest of 20 mite-susceptible and 20 mite-resistant colonies. At the same time, mite samples were collected from the infected pupae cells from 11 mite-susceptible and 10 mite-resistant colonies. All samples were immediately transported to the laboratory and stored at −80 °C until analysis.

Ten honey bees per colony were processed according to the method described by Antúnez et al. [43]. In the case of mites, 10 individuals per colony were subjected to mechanical homogenization in 50 uL of PBS using ceramic beads and a Fast Prep system (MP Biomedicals, Solon, OH, USA; 5 × 6.6 m/s for 30 s). Samples were centrifuged at 10,000 rpm for 5 min at 4 °C. In both cases (honey bees and mites), the supernatant was used for RNA extraction using a QIAamp Viral RNA Mini Kit (Qiagen, Germantown, MD, USA). Total RNA was subjected to reverse transcription using a QuantiTect Reverse Transcription Kit (Qiagen) according to the manufacturer’s conditions. Real-time PCR reactions were performed using a QuantiTec SYBR PCR Kit (Qiagen) and specific primers for the amplification of the following honey bee viruses: acute bee paralysis virus (ABPV) [44], deformed wing virus (DWV) [45], black queen cell virus (BQCV) [45], and sacbrood bee virus (SBV) [44]. For the normalization of the results, the expression level of the gene encodes for the honey bee β-actin was used [46].

Real-time PCR reactions were performed as described by Anido et al. [47] using a Rotor Gene 6000 (Corbett Research-Qiagen). The reaction mixture consisted of 1× QuantiTec SYBR Green PCR Master Mix, 0.5 µM of each primer (one pair of primers for each reaction), RNA-free water, and 5 µL cDNA in a final volume of 50 µL.

The cycling program consisted of an initial activation at 50 °C for 2 min and 95 °C for 15 min, and 45 cycles of 94 °C for 15 s, 50 °C for 30 s, and 72 °C for 30 s. Fluorescence was measured at the elongation step and controls without DNA were included in each reaction. The specificity of the reactions was checked by melting curve analysis of the amplified products (from 65 to 95 °C).

The amplified cDNA of each virus as well as that of the β-actin were expressed as the threshold cycle value (Ct). Ct value represents the number of cycles required to generate fluorescence that exceeds a predefined threshold. The threshold and reaction efficiency were calculated automatically using the Rotor-Gene 6000 software 1.7 (Corbett Research, Qiagen).

To control the variation in mRNA levels between the different samples, the data were normalized by subtracting the Ct value of β-actin from the Ct value of each virus (ΔCt). Subsequently, the viral load of each sample was estimated using the relative quantification method [48]. The concentration of all samples was analyzed with respect to the sample with the lowest viral load (“calibrator”); thus, the ΔCt of the calibrator was subtracted from the ΔCt of each sample (ΔΔCt = ΔCt of the sample −ΔCt of the calibrator). Finally, the value of 2^−ΔΔCt^ was calculated to estimate the relative levels of cDNA.

### 2.6. DWV Variants

cDNA obtained from honey bees and mites from 2 mite-susceptible and 2 mite-resistant colonies was subjected to qPCR-high-resolution melting (HRM) in order to amplify a 144 bp of the replicase gene [49]. Amplified fragments were cloned using a TOPO^®^ TA Cloning kit (Invitrogen), according to the manufacturer’s instructions. Ten clones per sample (8 samples in total) were sequenced at Macrogen (Seoul, Korea). The nucleotide sequences were compared to the GenBank database of the National Center for Biotechnology Information (Bethesda, MD, USA) using the BLAST tool.

Furthermore, cDNA obtained from honey bees of the 21 mite-susceptible and 21 mite-resistant colonies was amplified by qPCR in order to evaluate the presence of DWV variants A/B/C [50].

### 2.7. Molecular Characterization of Honey Bees

Five mite-susceptible and five mite-resistant colonies were selected and eight pupae per colony were collected and stored in 95% ethanol until analysis. Half of each pupa were processed individually and total DNA was extracted using the modified protocol of Miller et al. [51]. Five STR loci (i.e., A88, A113, A28, A43, and A9) were selected for PCR amplification and subsequent genotyping [52,53,54,55]. PCR amplification was performed in a PxE thermocycler (Thermo Electron Corporation, Milford, MA, USA). A total volume of 10 µL containing 4 ng/µL of template DNA was used for the reaction, 0.2 mM of each primer, 0.2 mM of each deoxynucleotide (dNTPs), 2 mM of MgCl2, 1× of enzyme buffer, and 0.5 units of Taq polymerase (Thermo Fisher Scientific, Waltham, USA). Initial denaturation was performed at 95 °C for 3 min, followed by a series of variable cycles according to the microsatellite to be amplified with denaturation at 94 °C for 30 s, between 51 °C and 60 °C during 30 s and 73 °C for 30 s, ending with 72 °C for 5 min. The success of the reaction was verified with electrophoresis on a 5% polyacrylamide gel visualized through silver nitrate staining [56]. The amplification products were processed in an ABI3500 Genetic Analyzer (Applied Biosystems, Foster City, CA, USA), with a DS-33 matrix and Liz600 as molecular weight standard. Peak Scanner 2.0 software (Applied Biosystems, Foster City, CA, USA) was used for individual genotype determination. Furthermore, genotypes were obtained from three reference populations (Europe, Brazil, and Africa) provided by Alice Pinto [57].

### 2.8. Molecular Characterization of V. destructor

Eight mites per colony were collected from five mite-susceptible and five mite-resistant colonies and kept in 95% ethanol until analysis. For DNA extraction, each mite was processed individually and total DNA was extracted using a previously described protocol [51]. Five loci of variable STRs were amplified (i.e., VD112, VD001, VD114, VD016, and VJ295) [58,59]. PCR amplification was performed in a Thermo PxE thermocycler, in a total volume of 10 µL containing 8 ng/µL of template DNA, 0.2 mM of each primer, 0.2 mM of each deoxynucleotide (dNTPs), 2 mM of MgCl2, 1× of reaction buffer, and 1 rc unit of Taq polymerase (Thermo Scientific). An initial denaturation was carried out at 95 °C for 3 min and, subsequently, a series of variable cycles according to the microsatellite to be amplified with denaturation at 94 °C for 30 s, between 55 °C and 62 °C for 30 s and 73 °C for 30 s, ending with 72 °C for 5 min. The success of the reaction was verified with electrophoresis on a 5% polyacrylamide gel visualized through silver nitrate staining [56]. Finally, the amplification products were processed in an ABI3500 Genetic Analyzer, with a DS-33 and Liz600 matrix as the molecular weight standard individually for each sample. Peak Scanner 2.0 software (Applied Biosystems, Foster City, CA, USA) was used for individual genotype determination.

### 2.9. Statistical Analysis

To compare adult population, brood area, hygienic behavior, grooming behavior, level of *V. destructor* infection in adult honey bees, fertility, abundance, prevalence, and intensity of *V. destructor* in brood cells, the ratio of mites on adult honey bees/mites in brood cells (adding 1 to the numerator and denominator to avoid having 0 values), and the viral load between mite-susceptible and mite-resistant colonies, the Wilcoxon test was used, as variables did not fit the assumptions of parametric statistics.

To test differences between *V. destructor* infection of drone and worker cells between mite-susceptible and mite-resistant colonies, a generalized linear model (GLM) analysis with a logit function was used. The response variable was presence (1) or absence (0) of mites and the predictor variables were cell type (i.e., drone or worker), population, and an interaction term between them. Model selection was done with an Akaike information criterion (AIC) [60,61], and the best fit for the data was achieved for the model with the lowest AIC value (∆AIC > 2).

The proportion of mite-susceptible and mite-resistant colonies infected by the four RNA viruses studied was compared using binomial tests to evaluate the presence of the virus in adult bees and mites.

All analyses were performed using the R statistical program (Vienna, Austria) [62]. The *p*-values lower than 0.05 were considered as statistically significant.

The genotypes obtained for each of the honey bee and mite samples were used to estimate population parameters. The allelic and genotypic observed and expected frequencies by Hardy–Weinberg equilibrium (HWE), the number of alleles (Na), observed (Ho) and expected (He) heterozygosity, and departures from HWE by exact test were estimated employing the GENEPOP v.4.1 package [63]. The genetic structuring was estimated with STRUCTURE 2.3.4 [64]. The program was instructed to test 1 to 10 K parameters using admixture ancestry model and correlated allele frequency for computing the Markov chain Monte Carlo (MCMC) simulation algorithm with a 10,000 burn-in length and a run length of 10,000. The simulation calculation was repeated 20 times for each K value. The K that best fit the data was chosen as the one that provided the highest likelihood values.

## 3. Results

### 3.1. Honey Bee Population and Brood Area

At the end of the summer, the population of honey bees in mite-susceptible and mite-resistant colonies was similar (10,010 ± 1540 and 9680 ± 2310 honey bees, respectively, W = 285.5; *p* = 0.297). In contrast, the brood area of mite-susceptible colonies was smaller than in the mite-resistant colonies (8096 ± 2816 and 10,560 ± 2464 cells, respectively, W = 121.5; *p* = 0.005).

None of the mite-susceptible colonies survived until the end of the autumn (June), whereas 82% of the mite-resistant colonies arrived in spring (September) in good condition.

### 3.2. Hygienic and Grooming Behaviors

**Figure 1:**
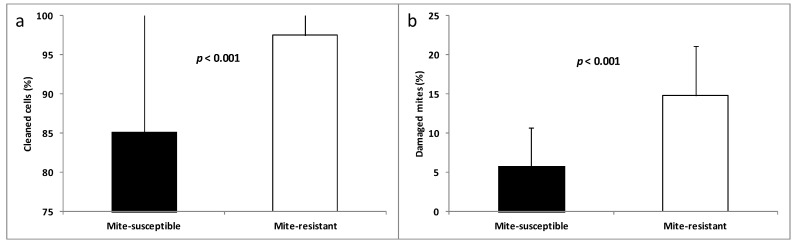
Hygienic (**a**) and grooming (**b**) behaviors in mite-susceptible and mite-resistant colonies.

Mite-resistant colonies displayed higher hygienic and grooming behaviors than the mite-susceptible colonies (Figure 1).

### 3.3. Mites in Honey Bees and Brood Cells

**Figure 2:**
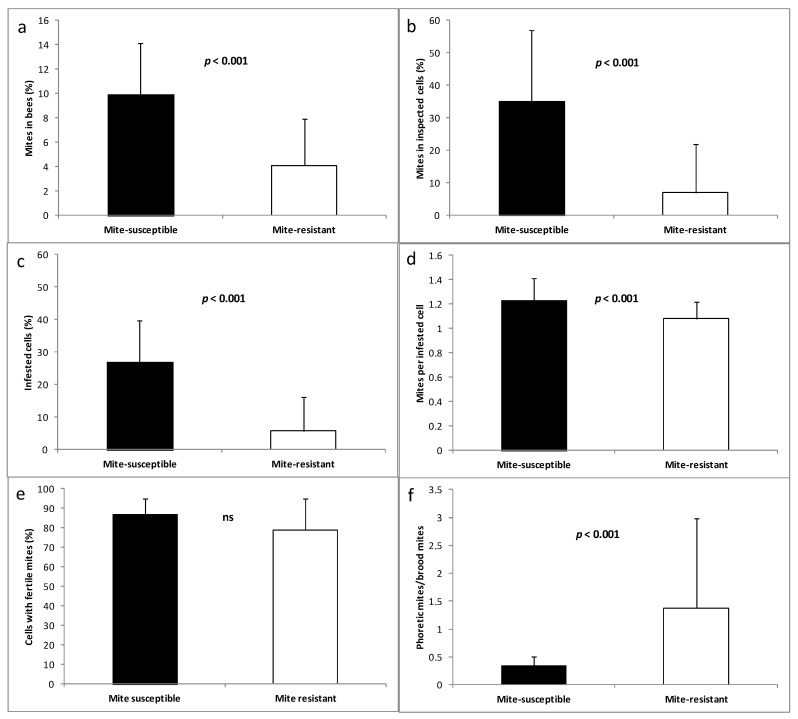
Presence of *V. destructor* in mite-susceptible and mite-resistant colonies. Infection in adult bees (**a**), abundance in brood cells (**b**), prevalence in brood cells (**c**), intensity of infection in brood cells (**d**), fertility (**e**), and relationship between phoretic and reproductive mites (**f**). ns: non significant.

At the end of the summer, mite-susceptible colonies showed more mites in honey bees and in the brood (reflected in values of abundance, prevalence, and intensity of infection), as well as a higher proportion of phoretic mites/mites in brood, than mite-resistant colonies. In contrast, the fertility of *V. destructor* was similar in both apiaries (Figure 2).

**Figure 3:**
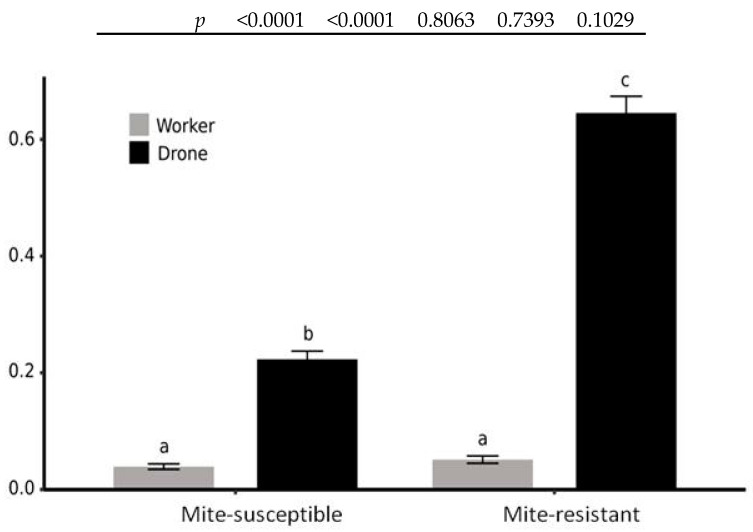
Estimated probability of *V. destructor* infestation in drone and worker cells in mite-susceptible and mite-resistant colonies according to generalized linear model (GLM) analysis with link logit. Different letters show significance level (*p* < 0.05) based on the odds ratio confidence intervals estimated in the model.

Regarding the preference of the mites for the worker and drone cells, the best model explaining mite infection included all predictor variables (cell type, apiary, and the interaction between them) (Table 1 and Table 2). Drone cells of mite-resistant colonies are three times more likely to be infected by the mite than those of mite-susceptible colonies. In contrast, worker cells have a low probability of being infected with no differences found between the two apiaries (Figure 3).

### 3.4. RNA Viruses in Honey Bees and Mites

**Figure 4:**
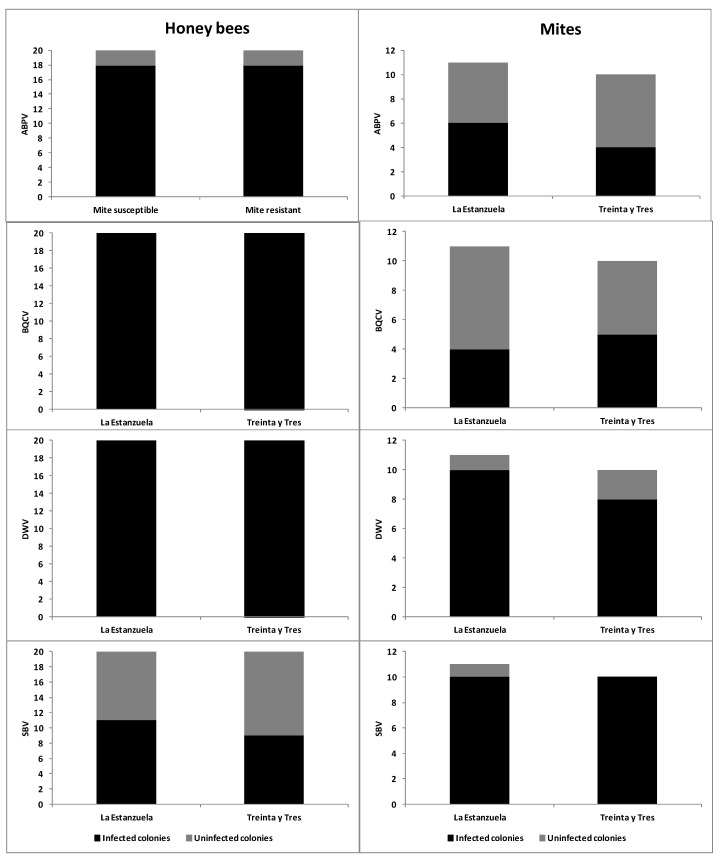
Percentage of colonies presenting honey bees or mites infected with acute bee paralysis virus (ABPV), black queen cell virus (BQCV), deformed wing virus (DWV), and sacbrood bee virus (SBV) in mite-susceptible and mite-resistant colonies.

The four analyzed viruses (ABPV, BQCV, DWV, and SBV) were detected in mite-susceptible and mite-resistant colonies, both in adult honey bees and in mites. BQCV and DWV were detected in honey bees from all colonies in the two apiaries (Figure 4). No statistical differences (*p* > 0.10) were found in the proportion of colonies infected by the four viruses studied between both groups, considering the presence of the viruses in bees and mites (Figure 4).

The infection level of ABPV, BQCV, and SBV was similar in mite-susceptible and mite-resistant colonies (*p* > 0.10). However, in the case of DWV, mite-susceptible colonies showed a higher infection level than mite-resistant colonies (relative DWV level 2467 ± 5784 and 588 ± 2406, respectively, W = 294; *p* = 0.011).

Mites of colonies from both apiaries showed similar viral loads of the four analyzed viruses (*p* > 0.10).

According to the results of qPCR-HRM, clone sequencing, and A/B/C qPCR, only DWV variant A was detected in mites and honey bees from mite-susceptible and mite-resistant colonies.

### 3.5. Molecular Characterization of Honey Bees

The number of alleles found for each locus in the two honey bee populations is shown in Table 2. In all observed cases, heterozygosis was significantly lower than expected in all the studied loci (Table 2). This was not found in the reference populations. A lower number of alleles was recorded for Uruguayan samples than for references.

**Figure 5:**
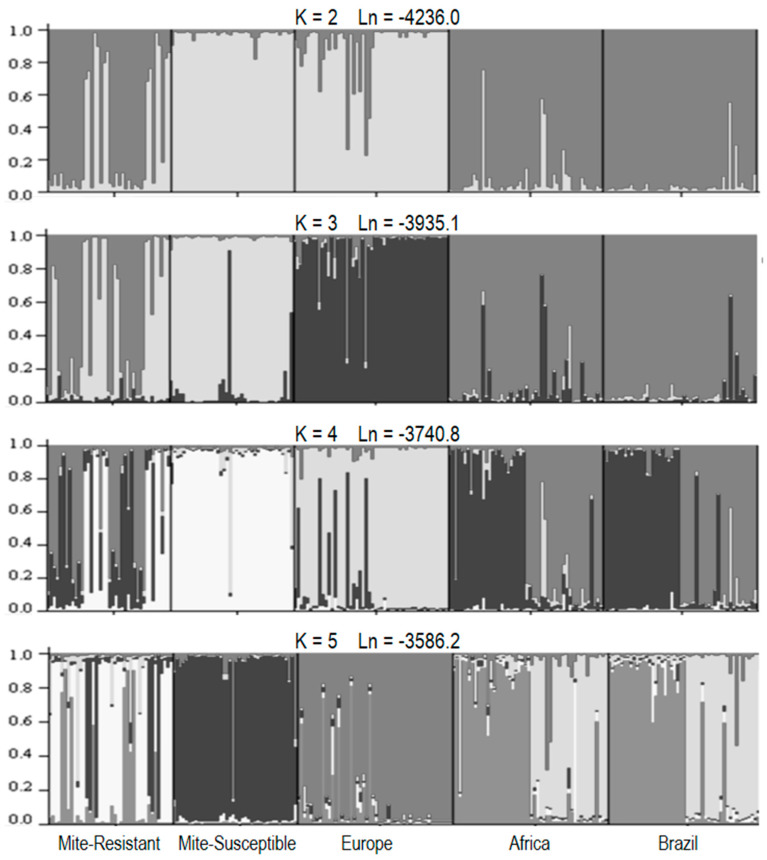
Honey bee population allocation by the STRUCTURE program based on the genotyping of 5 STR loci. Each individual is represented by a partitioned vertical bar, with partitions (represented by different colors) representing the posterior probability of each individual belonging to one or another population. K: number of populations assumed by the model; Ln: average likelihood value for all runs for the same K.

The distribution of allelic and genotypic frequencies was significantly different between mite-susceptible and mite-resistant colonies (*p* < 0.0001 in both cases). The differences between the two studied populations and between them and the reference populations can be graphically visualized in the figures generated by the STRUCTURE 2.3.4 software [64], which evaluates the combined variation of all microsatellites and assigns individuals to different theoretical populations. The model that better adjusts when analyzing only the samples from the two Uruguayan apiaries is that of two populations, coinciding with the samples from mite-resistant and mite-susceptible colonies, respectively (Figure S1, Supplementary Materials). When the reference population samples are included in the analysis (Figure 5), the model that fits better is the five-population model (K = 5), i.e., mite-resistant colonies, mite-susceptible colonies, Europe, and two hypothetical populations of mixed composition, with individuals from Brazil and Africa in similar proportions. When grouping into four populations (K = 4), these two hypothetical populations also appear, but include individuals from Africa, Brazil, and mite-resistant colonies. Some of the mite-resistant honey bees showed great affinity with mite-susceptible honey bees. In the model with three populations (K = 3), these hypothetical populations merge and form a single population made up of all individuals from Brazil and Africa, plus many individuals from mite-resistant honey bees. Again, some mite-resistant individuals showed greater affinity with the mite-susceptible individuals. The simplest grouping occurs when adjusting for two hypothetical populations (K = 2). In this case, one population is integrated by mite-resistant colonies, Brazil, and Africa, whereas the other corresponds to samples from mite-susceptible colonies and Europe. This model, while not being the best adjusted, shows the genetic affinities of honey bees from both Uruguayan apiaries with respect to those of reference samples.

### 3.6. Molecular Characterization of V. destructor

The number of alleles found for each locus in the two mite populations ranged between 1 (VD016 monomorphic locus) and 4 (Table 3). In all studied loci, observed heterozygosis was lower than expected, and there was a very significant deviation from the HWE (Table 3).

When comparing allelic and genotypic frequencies of mite populations of mite-resistant and mite-susceptible colonies, significant differences were found for the VJ295 locus (*p* = 0.0006 and *p* = 0.0014 for the allelic and genotypic frequencies, respectively). For the VD112 locus, marginal differences were found for allelic frequencies (*p* = 0.0534) and significant differences for genotypic frequencies (*p* = 0.0014). On the contrary, for VD001 and VD114 loci, no significant differences in allelic and genotypic frequencies were found between the two populations of mites studied (*p* > 0.10 in all cases).

## 4. Discussion

In Uruguay, after 40 years of interaction between honey bees and *V. destructor*, colonies in most of the country need acaricides to survive. A different scenario occurs in the eastern side of the country where the mite does not cause significant problems. The exhaustive analysis carried out of the factors that could explain the notable differences found in the *A. mellifera*–*V. destructor* relationship in two Uruguayan regions showed that this relationship is complex.

In the first place, this study confirmed the resistance to *V. destructor* of the honey bee population from the eastern side of the country, which was able to survive without acaricide treatment and showed only 18% colony mortality. In contrast, the western honey bee population showed extreme susceptibility to the mite, since none of the colonies managed to overcome autumn.

### 4.1. Resistance Behaviors to V. destructor

Behavioral resistance of honey bees appears as a critical factor in controlling the *V. destructor* population. *Varroa destructor*-resistant colonies showed higher hygienic behavior than mite-susceptible colonies. Numerous studies indicate that hygienic colonies display a better control of the *V. destructor* population [21,65,66,67]. Nevertheless, the role of hygienic behavior in limiting mite reproduction is still controversial [68,69].

The hygienic behavior exhibited by honey bees that are able to detect the onset of *V. destructor* reproduction and uncap the cell containing the infected pupae is called Varroa Sensitive Hygiene (VSH) [21]. A recent study, in which brood was artificially infected with *V. destructor*, showed that honey bees from Treinta y Tres presented higher VSH than honey bees from La Estanzuela (Alexis Beaurepaire, unpublished data). The interruption of *V. destructor* reproduction by hygienic honey bees, especially if the mites have already laid eggs, leads to a decrease in the mite population due to lost opportunities to reproduce [70,71].

Hygienic behavior may play a key role in honey bee populations with natural resistance to *V. destructor*, such as those from Brazil and South Africa. However, other factors explain mite resistance in honey bees from Fernando de Noronha island (Brazil) or those from Gotland island (Sweden) [13]. Recently, Oddie et al. [72] compared four populations of naturally surviving *V. destructor* bees with populations of susceptible local honey bees. They found that resistant honey bees uncap infested brood cells with higher frequency, and then recap the cells without the need to remove pupae. This behavior, a product of rapid evolution, avoids the cost of losing brood. These results were confirmed by Martin et al. [73] when comparing cell recapping in mite-resistant honey bees from Brazil and Africa (*A. m. scutellata*) with “mite-naive” honey bees from the United Kingdom and Australia.

Grooming behavior could also contribute to the better resistance to *V. destructor* presented by colonies from Treinta y Tres since this behavior was better expressed than in mite-susceptible colonies from La Estanzuela. In Uruguay, Invernizzi et al. [41] found that Africanized bees expressed more grooming than European bees (*A. m. ligustica*), both at colonial and individual levels. This behavior has already been reported as a valuable trait for the control of *V. destructor* [22,23,24,25]. However, we must be cautious when evaluating the importance of grooming behavior as resistance to *V. destructor* since a significant number of mites collected on the floor come from brood cells [74,75]. It is likely that part of the damaged mites in mite-resistant honey bees originated from the cleaning of the parasitized cells (hygienic behavior).

The differences in the expression of the two studied resistance behaviors could explain the lower infestation level by *V. destructor*, both in honey bees and brood, that the mite-resistant colonies presented when compared to mite-susceptible colonies at the beginning of the autumn. The selection of honey bees possessing these traits appears as a promising alternative to improve the resistance of honey bees to varroosis [18,19]. In this sense, progress has been made in the knowledge of genes associated with both behaviors, which would allow selection based on molecular markers [25,76,77].

### 4.2. Reproductive Aspects of V. destructor

The *V. destructor* fertility in both honey bee populations was similar, indicating that the difference in the mite population in both apiaries is not due to reproductive differences. This result contrasts with other studies that relate *V. destructor* fertility to the growth of mite populations in the colonies [23,69,78,79]. However, it cannot be ruled out that, with a larger sample, small differences would appear in the fertility of the mites of the two populations, having an impact on the level of infection in the colonies.

The phoretic mite/reproductive mite ratio was four times higher in mite-resistant colonies than in mite-susceptible colonies. This vital difference could be explained by the more remarkable hygienic behavior displayed by honey bees from the first apiary that allows interrupting the mites’ reproduction in the cells, eliminating or forcing them to enter into the phoretic phase [72,80]. Whatever the fate of the mites found in the uncapped cells, the consequence is an increase in the phoretic mite population at the expense of the reproductive ones.

Another difference between both groups of colonies was the ratio of mites present in the drone and worker cells, since the probability of infecting drone cells was 3-fold greater in mite-resistant than in mite-susceptible colonies. *Varroa destructor* in its primary host, the Asian honey bee *A. cerana*, reproduces almost exclusively in drone cells [4,5]. Nevertheless, in *A. mellifera*, the mite maintains the preference to reproduce in drone cells but also in worker cells [1]. The preference ratio into the two cell types ranges from 12:1 to 8:1 [81,82,83]. The values found in mite-resistant colonies were similar to those mentioned (12.6:1), whereas in mite-susceptible colonies this ratio decreases markedly (5.7:1). This relationship has been poorly analyzed, although it may have significant consequences for the colonies’ survival. If the worker population is harmed by increased mite reproduction into their cells, the colony viability and its chances of reproduction are compromised. In this sense, it has been found that the parasitization of worker pupae by *V. destructor* decreases honey bees’ longevity [84,85] as well as their weight and flight capacity [86,87], increases the viral load of DWV [7,8], and suppresses the immune response exposing honey bees to infection by other organisms [6]. At the colony level, infected colonies produce fewer swarms [88,89]. A different virulence is possible among populations of *V. destructor* associated with its ability to reproduce in worker cells. This change in the reproductive biology of the mite may be a consequence of the colonies’ density. Dynes et al. [16] tested the evolutionary hypothesis that mites from densely beekeeper-managed colonies would be more virulent than those from wild colonies (which rely more on vertical transmission to spread). When comparing the growth of the mite population of the two origins, the mites from wild colonies reproduced more slowly than those from commercial colonies. In line with this argument, it is essential to note that, in the Treinta y Tres Department (the location of the mite-resistant colonies), beekeeping activity is poorly developed and the density of colonies is 1 colony/km^2^. At the same time, in the Colonia Department (the location of the mite-susceptible colonies), there are many beekeepers and the density of colonies is 10 colonies/km^2^.

### 4.3. Presence of Viruses in Honey Bees and Mites

In both honey bees and mites from mite-resistant and mite-susceptible colonies, the viruses ABPV, BQCV, DWV, and SBV were found. The BQCV and DWV were present in honey bees from all colonies in both apiaries. There were no significant differences in the proportion of colonies with mites or honey bees infected by each virus in both honey bee populations. In addition, there were no significant differences between apiaries regarding the viral load of ABPV, BQCV, and SBV in mites and honey bees. However, mite-resistant honey bees showed a lower infection level by DWV than mite-susceptible colonies. It is possible that behavioral resistance to *V. destructor* shown by mite-resistant colonies limits their population and indirectly reduces DWV replication, preventing colony collapse. According to de Miranda and Genersch [8], the probability of pupae getting infected by DWV increases according to the *V. destructor* population. This situation can lead to the collapse of colonies. Similar results to those found in this study were obtained by Emsen et al. [90] comparing the load of several viruses in selected colonies for high and low growth of *V. destructor* population, finding that honey bees with higher resistance to the mite had less DWV load than the susceptible honey bees. In contrast, Locke et al. [91], who compared the evolution of RNA viruses in *V. destructor*-resistant honey bees (Gotland island population) to those in susceptible ones from summer to winter, found that DWV was the same in the two groups of colonies. Those authors suggest that *V. destructor*-resistant honey bees would have greater tolerance to DWV.

Regarding DWV variants, variant A was the only variant detected in both honey bee and mite populations. Martin et al. [92] showed that varroa facilitates the dominance of certain DWV strains, decreasing the viral diversity. In this sense, the low DWV genetic diversity detected is consistent with the long establishment of *V. destructor* in Uruguay. DWV variant A was also the dominant variant in the region, including Chile [93], Brazil [94], and Argentina [95], and seems to be more virulent than DWV-B at the colony level. However, this point is under discussion, since other studies showed that variant DWV-B was more virulent [96,97].

### 4.4. Genetic Differences between Honey Bees

Microsatellite analysis in honey bees showed very marked allelic and genotypic differences between both populations. Some samples from Treinta y Tres had a higher posterior probability of belonging to the La Estanzuela honey bee population, indicating a certain degree of genetic exchange between both populations, undoubtedly due to the colony movement carried out by beekeepers. When incorporating the reference samples from Africa, Brazil, and Europe into the analysis [57] and asking STRUCTURE to form two groups (K = 2), clearly the honey bees of Treinta y Tres cluster with those from Brazil and Africa, and those from La Estanzuela with the European sample. Although 80% of honey bees in Uruguay belong to the African haplotype of *A. m. scutellata* [36], Africanization degree can still have a gradient from north and east to southeast, as described by Diniz et al. [35]. The genetic similarity of La Estanzuela honey bees to European honey bees can also be explained by the strong commercialization of European queens (especially *A. m. ligustica*) since beekeepers in the region highly value the gentleness of this subspecies. In contrast, in the eastern part of the country, the predominant honey bee populations are the Africanized ones that prevail in Brazil and the region [98,99]. When the program tested five groups, it was found, as expected, that the five analyzed bee populations were separated.

The fact that the two studied honey bee populations display genetic differences at the subspecies scale (the *V. destructor*-resistant populations of Treinta y Tres show a higher degree of Africanization, whereas the La Estanzuela colonies are more susceptible to the mites and more European-like) indicates that the differential resistance found to varroosis may reflect, at least in part, the resistance of each honey bee subspecies. Africanized honey bees are widely known to show good resistance to *V. destructor*, possibly due to greater hygienic and grooming behaviors than those of European honey bees [13].

### 4.5. Genetic Differences between Mites

The microsatellite analyses showed that the two populations of *V. destructor* displayed significant differences in allelic and genotypic frequencies. Until a few years ago, the population of *V. destructor* was thought to be almost genetically uniform, regardless of the analyzed regions [31]. The differences found coincide with recent studies that show significant genetic variability in *V. destructor* populations [32,33]. These results indicate that differences in the reproductive behavior of *V. destructor* between the two studied apiaries (ratio between phoretic and reproductive mites, and preference to reproduce in drone or worker cells) may be associated with genotypic variants. This is an aspect that will have to be studied in the future to understand the different *A. mellifera*–*V. destructor* relationships found worldwide.

### 4.6. Final Considerations

This study showed that the behavioral resistance of honey bees (hygienic and grooming behaviors) to *V. destructor* is critical to controlling mite populations. It is possible that the control of the *V. destructor* population reduces the DWV load in honey bees, the virus most associated with the mite, mitigating the damage it causes. The differences found in behavioral resistance may be associated with genetic differences in honey bees at the subspecies level. In any case, some aspects of the reproductive biology of *V. destructor* could be affecting the damage that this parasite causes to the colonies. These differences could be associated with genetic variants of the mite. The identification of a population of honey bees with clear resistance to *V. destructor* in Uruguay is an addition to those reported in other countries and contributes to the search for tools to improve mite control.

## Figures and Tables

**Table 1:** Indicators of the level of infection by *V. destructor* in the totality of inspected drone cells and worker cells in 8 mite-susceptible colonies and 5 mite-resistant colonies.

Colonies		Mite-Susceptible	Mite-Resistant
Drones	Inspected cells	887	282
	Mite-infested cells	176	101
	Total mites	198	182
	Prevalence	19.8%	35.8%
	Abundance	22.3%	64.5%
Workers	Inspected cells	1710	1210
	Mite-infested cells	67	60
	Total mites	67	62
	Prevalence	3.9%	5.0%
	Abundance	3.9%	5.1%
Ratio of mite distribution between drone and worker cells		5.70	12.60

**Table 2:** Number of analyzed individuals (N), number of alleles found (Na), expected heterozygosity (He), observed heterozygosity (Ho), and probability of departure from Hardy–Weinberg equilibrium (*p*) for each microsatellite in mite-resistant honey bee populations of Treinta y Tres (Mite-R), mite-susceptible honey bee populations of La Estanzuela (Mite-S), and the reference honey bee populations from Europe (EU), Africa (AF), and Brazil (BR).

Locus		Mite-R	Mite-S	EU	AF	BR
A43	N	32	22	50	41	32
	Na	9	7	6	16	13
	Ho	0.063	0.682	0.640	0.854	0.875
	He	0.769	0.766	0.615	0.881	0.851
	*p*	<0.0001	<0.0001	0.2545	0.4865	0.9758
A88	N	32	23	50	41	32
	Na	7	3	6	14	12
	Ho	0.125	0.043	0.700	0.878	0.750
	He	0.771	0.463	0.629	0.878	0.853
	*p*	<0.0001	<0.0001	0.6378	0.8287	0.1182
A28	N	31	39	50	41	32
	Na	10	2	2	9	10
	Ho	0.065	0.000	0.380	0.805	0.813
	He	0.803	0.099	0.413	0.833	0.806
	*p*	<0.0001	<0.0001	0.2363	0.4356	0.4059
A8	N	38	37	50	41	32
	Na	8	4	6	9	9
	Ho	0.211	0.081	0.800	0.829	0.688
	He	0.780	0.681	0.801	0.838	0.802
	*p*	<0.0001	<0.0001	0.0825	0.6681	0.0440
A113	N	34	39	50	41	32
	Na	9	8	11	12	11
	Ho	0.382	0.769	0.640	0.854	0.875
	He	0.788	0.799	0.650	0.858	0.858
	*p*	<0.0001	<0.0001	0.8063	0.7393	0.1029

**Table 3:** Number of analyzed individuals (N), number of alleles found (Na), expected heterozygosis (He), observed heterozygosis (Ho), and probability of departure from Hardy–Weinberg equilibrium (*p*) for each STR loci in populations of mites from mite-resistant colonies of Treinta y Tres (Mite-R) and mite-susceptible colonies of La Estanzuela (Mite-S).

Locus		Mite-R	Mite-S
VD112	N	29	33
	Na	2	3
	He	0.068	0.222
	Ho	0.000	0.242
	*p*	0.0175	1.000
VD001	N	29	26
	Na	3	2
	He	0.101	0.075
	Ho	0.034	0.000
	*p*	0.0175	0.0196
VD114	N	28	33
	Na	1	2
	He	0.000	0.060
	Ho	0.000	0.000
	*p*	NA	0.0154
VD016	N	29	30
	Na	1	1
	He	0.000	0.000
	Ho	0.000	0.000
	*p*	NA	NA
VJ295	N	28	28
	Na	4	3
	He	0.546	0.450
	Ho	0.500	0.500
	*p*	0.4031	0.0618

## Supplementary Materials

The following are available online at https://www.mdpi.com/2306-7381/7/3/116/s1, Figure S1: Honey bee population allocation by the Structure program based on the genotyping of 5 STR loci.
