# Supplementary material for: Unraveling Honey Bee–Varroa destructor Interaction: Multiple Factors Involved in Differential Resistance between Two Uruguayan Populations
Source: Vet Sci. 2020 Aug 20;7(3):116. doi: 10.3390/vetsci7030116 (PMC7558146; doi:10.3390/vetsci7030116)
Supplement: Supplementary file 1 [file vetsci-07-00116-s001.pdf]

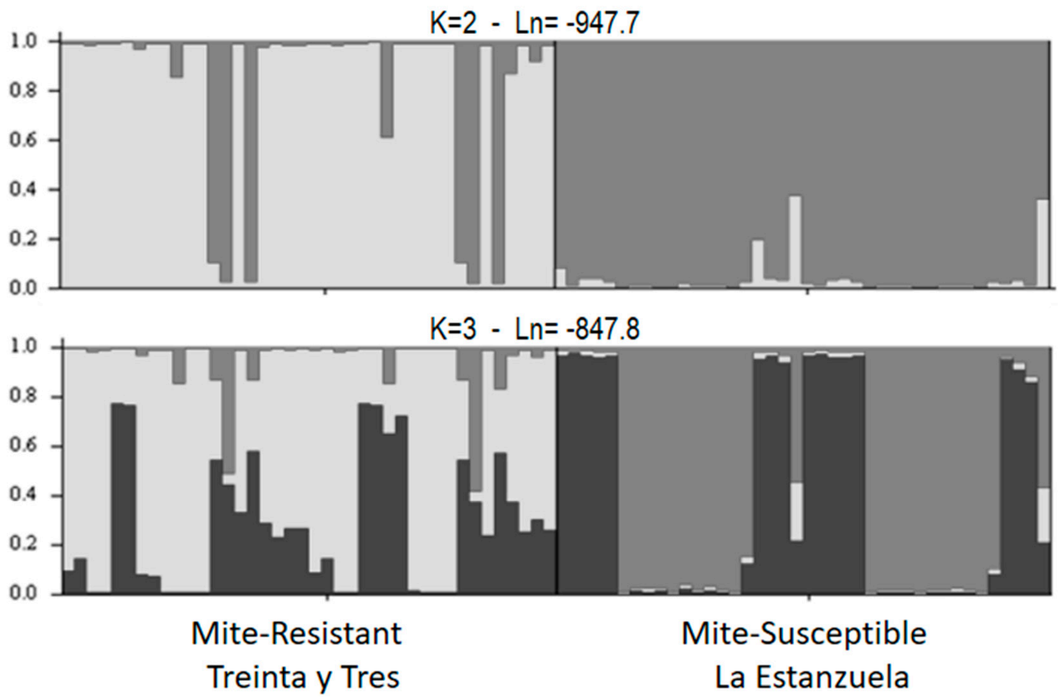

Figure S**1.** Honey bee population allocation by the STRUCTURE program based on the genotyping of 5 STR loci. Each individual is represented by a partitioned vertical bar, with partitions (represented by different colors) representing the posterior probability of each individual belonging to one or another population. K: number of populations assumed by the model; Ln: average likelihood value for all runs for the same K.
